# (A)Symmetric Stem Cell Replication and Cancer

**DOI:** 10.1371/journal.pcbi.0030053

**Published:** 2007-03-16

**Authors:** David Dingli, Arne Traulsen, Franziska Michor

**Affiliations:** 1 Program for Evolutionary Dynamics, Harvard University, Cambridge, Massachusetts, United States of America; 2 Division of Hematology, Mayo Clinic College of Medicine, Rochester, Minnesota, United States of America; 3 Society of Fellows, Harvard University, Cambridge, Massachusetts, United States of America; 4 Dana Farber Cancer Institute, Boston, Massachusetts, United States of America; University of Auckland, New Zealand

## Abstract

Most tissues in metazoans undergo continuous turnover due to cell death or epithelial shedding. Since cellular replication is associated with an inherent risk of mutagenesis, tissues are maintained by a small group of stem cells (SCs) that replicate slowly to maintain their own population and that give rise to differentiated cells. There is increasing evidence that many tumors are also maintained by a small population of cancer stem cells that may arise by mutations from normal SCs. SC replication can be either symmetric or asymmetric. The former can lead to expansion of the SC pool. We describe a simple model to evaluate the impact of (a)symmetric SC replication on the expansion of mutant SCs and to show that mutations that increase the probability of asymmetric replication can lead to rapid mutant SC expansion in the absence of a selective fitness advantage. Mutations in several genes can lead to this process and may be at the root of the carcinogenic process.

## Author Summary

In multicellular organisms, tissues such as skin, the gut, and blood undergo continuous cell turnover. These tissues are maintained by a small group of tightly regulated cells known as stem cells (SCs) that have two defining properties: they can renew themselves and give rise to more specialized cells that perform tissue specific tasks. Somatic SCs live for many years and replicate slowly to minimize the risk of acquiring mutations in their DNA. When a SC divides, the two daughter cells may have similar properties (symmetric division) or may have different fates (asymmetric division). Symmetric division may allow SCs to expand, and mutations that alter the probability of symmetric versus asymmetric division might increase the risk of tumor growth. This property is important since there is increasing evidence that even tumors have their own SCs. Mutations can transform wild-type SCs into tumor SCs with modified cell division properties, which have decisive impact on cancer progression. Here we develop a mathematical model to illustrate the impact of mutations that regulate the symmetry of SC division on the development of tumors. Our results provide novel insights on the pathway to cancer by mutations within SCs.

## Introduction

Most tissues of the body experience continuous cell turnover due to either apoptosis or loss of epithelial cells due to shedding as occurs in the gastrointestinal and respiratory tract as well as the ductal epithelium of the breast. This cell turnover also gives tissues the ability to repair after injury. Cell replication is associated with an inherent risk of mutation due to the imperfect replication machinery [1]. Cancer is due to the progressive accumulation of mutations [2], and to minimize the risk of malignant transformation, higher vertebrates have evolved tissue architecture so as to limit the retention of cells that acquire mutations [3–5]. In general, tissues are maintained by a small group of slowly replicating cells that have the dual capacity of self-renewal and differentiation into the more mature cells required by a given tissue. Cells that exhibit these two capabilities are known as stem cells (SCs).

The SC concept was initially proposed for cells responsible for the maintenance of hematopoiesis [6–8]. Hematopoietic SCs are located within the bone marrow, and their self-renewal capacity has classically been demonstrated by an ability to reconstitute hematopoiesis after serial transplantation in sublethally irradiated mice [9,10]. Recent studies have shown that SCs are present in most tissues of the body including the colonic mucosa, the breast, and the central nervous system [11]. Moreover, it is increasingly recognized that many tumors have a population of cancer stem cells (CSCs) that maintains the growth of the neoplasm [7,11–16]. Eradication of the CSCs is necessary for therapeutic cure of the neoplasm [17].

A critical aspect of SC replication is the diverse fate of the two daughter cells that result from each division. Conceptually, such divisions are considered to be symmetric if the two daughter cells are identical to each other (whether they both retain SC properties or become committed cells), or asymmetric if one daughter cell retains the SC properties of the parent while the other cell commits to a more differentiated stage (Figure 1) [18]. In principle, asymmetric replication within the SC pool can maintain cellular homeostasis since one daughter cell is retained as an SC while the other enters a committed pathway. However, SCs have to be able to replicate symmetrically to generate two daughter SCs, since this is the only way the SC pool may expand during ontogeny or after injury such as SC transplantation [18,19]. An SC may divide symmetrically into two committed daughter cells, and therefore an SC is “lost” from the pool, whereby a symmetric division in which the daughter cells remain as SCs again becomes necessary. Studies on the methylation patterns of DNA in daughter cells provide experimental evidence for all three types of SC division in colonic crypts [20], and recent experiments in animal models with serial hematopoietic SC transplantation also support the existence of all three types of division [19].

**Figure 1:**
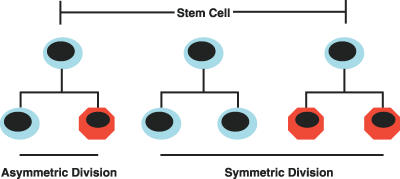
SC Replication Can Give Rise to Daughter Cells That Retain the Properties of the SCs or Become Committed to Differentiate If the two daughter cells share the same fate, the preceding division is considered to be symmetric; while if one daughter cell retains SC capabilities and the other differentiates, the preceding mitotic event is considered to be asymmetric. Normal SCs are in blue and differentiated cells are depicted in red.

The mechanisms regulating the (a)symmetry of SC divisions are not completely understood. However, it appears that both interactions with the SC niche as well as intracellular mechanisms relating to the mitotic apparatus and differential partitioning of intracellular organelles contribute to the different modes of replication of these cells [18]. Studies in model organisms such as Drosophila melanogaster and Caenorhabditis elegans are beginning to provide a mechanistic understanding of the various determinants of symmetric versus asymmetric SC division [18]. The dynamic implications of symmetric versus asymmetric SC replication have been modeled only indirectly [21,22], even though this is an important area of investigation since mutations in tissue-specific SCs may be the source of many neoplasms [11,23,24]. Here we develop a simple mathematical model of SC dynamics that only considers the probability of symmetric versus asymmetric replication within the SC compartment to illustrate the potential consequences of disrupted SC division.

## Methods

### The Model

Consider a microenvironment composed of a limited number of SCs; an example is the colonic crypt that houses intestinal SCs [25]. SCs divide to maintain themselves or differentiate into the colonic epithelial cells that migrate up the crypt where they undergo apoptosis and are either shed into the lumen or engulfed by stromal cells [25–28]. The number of SCs remains approximately constant (approximately one to ten SCs per crypt). Therefore, SC dynamics in the crypt can be modeled by a Moran process [29], which is a stochastic process assuming that the total population size remains strictly constant over time (Figure 2). Denote the total number of SCs in a given crypt by *n*. Consider the fate of a single mutant SC: it can either go extinct or reach fixation in the population. The evolutionary dynamics of the SC population as a function of symmetric and asymmetric replication is modeled as follows.

**Figure 2:**
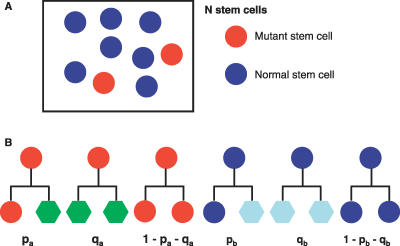
Representation of the Stochastic Model of Mitotic SC Replication The model is based on the Moran process, which maintains a constant population size (A) and in which cells are chosen for reproduction proportional to their fitness. (B) Normal (blue) and mutant (red) SCs may divide asymmetrically with probability *p,* while they divide symmetrically with probability *q* to produce two differentiated cells. SCs also can divide symmetrically to produce two daughter SCs with probability 1 − *p* − *q*.

Denote by *i*. the number of mutant SCs; therefore, the number of normal SCs is given by *n* − *i*. The relative reproductive fitness of normal SCs is 1 while that of the mutant SC is given by *r*. If *r* < 1, mutant cells have a lower fitness as compared with normal SCs, while if *r* = 1 the mutant SCs have the same fitness as normal SCs. If *r* > 1, the mutant cells have a higher reproductive fitness compared with the normal SC. During each replication cycle, SCs are randomly chosen for reproduction proportional to their fitness. If an SC divides symmetrically to produce two daughter SC, then one SC from the whole pool is selected at random to be eliminated so that the total number of SC, *n,* remains strictly constant. If the SC selected for reproduction gives rise to two differentiated daughter cells, one SC is effectively lost from the compartment. To maintain SC homeostasis in that population, another SC is selected (again according to fitness) for reproduction and gives rise to two daughter SCs so that the total number of SCs is restored. Finally, the selected SC may divide asymmetrically to give rise to one differentiated cell and another SC, whereby the number of SCs in the compartment remains constant. Therefore, with each replication event there are three potential scenarios for both mutated and normal SCs (Figure 2). The number of mutants can increase by one, stay the same, or decrease by one. We denote by *p_a_* the probability that the mutant SC divides asymmetrically, while *q_a_* is the probability of a symmetric replication that produces two differentiated cells. Finally, the probability that the mutant SC divides symmetrically to give two daughter SCs is given by 1 − *p_a_* − *q_a_*. The same nomenclature with subscript *b* denotes the respective division probabilities for the normal SC (Figure 2).

Let us consider the transition probabilities of the stochastic process. For the mutant SC population to increase by one, a mutant SC must be selected for reproduction, and a normal SC selected for death. Alternatively, a normal SC may divide symmetrically to produce two differentiated daughter cells, and a mutant SC is selected for reproduction where the cell has to reproduce itself. The transition probability therefore is given by

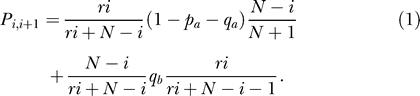



Similarly, two separate paths decrease the mutant SC number by 1 in a unit time step. A normal SC may reproduce itself, and a mutant SC is selected for death. Otherwise, a mutant SC may reproduce to give rise to two differentiated cells, and a normal SC is selected for self-renewal. The transition probability for this process is given by

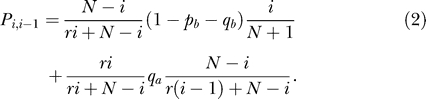



Finally, the probability that the number of mutant SCs remains constant is given by

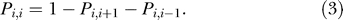



To calculate the probability that *i* mutant SCs ultimately take over the population, we observe that this fixation probability Φ*_i_* follows the equation

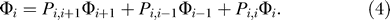



Iterating this equation leads to the solution

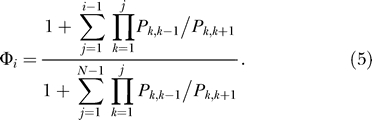



See, e.g., Antal and Scheuring [30] for details of the derivation. Here *P_k,k_*_ + 1_ and *P_k,k_*_−1_ are as defined in Equations 1 and 2, respectively. The fixation probability of a single mutant in the SC population is given by

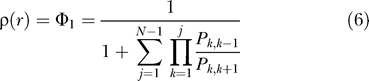



It can be shown that if *p_a_* = *q_a_* = *p_b_* = *q_b_* = 0, the process is identical to the classical Moran process [29] with 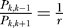 and 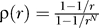 .

The conditional average time τ*_i_* until a population of *i* mutant CSs reaches fixation follows the equation

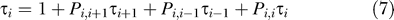



Starting with zero mutant cells, fixation is never reached, and hence τ_0_ = 0. If we start from *n* mutant cells, fixation has already occurred, and therefore τ*_n_* = 0. With these boundary conditions, we can solve Equation 7. The average conditional time required for a single mutated cell to take over the SC pool is given by

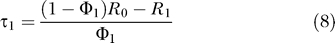
where

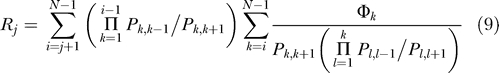



## Results

We performed exact stochastic computer simulations for different parameter regimes. We varied both the fitness *r* of the mutant SC population as well as the probabilities for symmetric versus asymmetric reproduction to show the time development of the process. Initially, we explored the model for *n* = 1,000. We chose this value for *n* since it is unlikely that any SC compartment is composed of a larger number of active SCs [31]. In the absence of a mutated SC, the number of normal SCs remains constant (Figure 3A). We set the baseline transition probabilities for the normal SC as *p_b_* = *q_b_* = 1/3, so that the probability for each type of mitotic division is equal.

**Figure 3:**
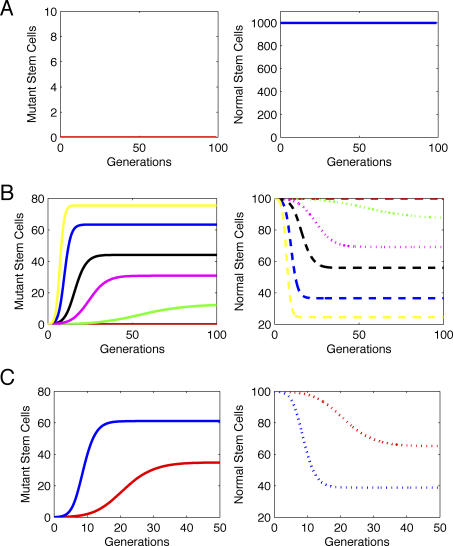
SC Dynamics (A) In the absence of mutant SCs, normal SC homeostasis is maintained in the stochastic process, and the abundance of normal SCs, *n* = 1,000, remains constant. Transition probabilities are *p_b_* = *q_b_* = 1/3. (B) If a neutral mutant SC appears (i.e., a cell with the same reproductive fitness as a normal SC), random drift may allow the population to expand and take over the SC compartment (red) (*p_a_* = *p_b_* = *q_a_* = *q_b_* = 1/3, *a*_0_ = 1, b_0_ = 999, *r* = 1, averaged over 1,000 runs and the percentage plotted). As the probability for self-renewal of the mutant SC increases, a higher fraction of compartments are taken over by the mutant SC (green, *p_a_* = *q_a_* = 0.3; magenta, *p_a_* = *q_a_* = 0.25; black, *p_a_* = *q_a_* = 0.2; blue, *p_a_* = *q_a_* = 0.1; yellow, *p_a_* = *q_a_* = 0). (C) A mutation that increases both the probability of self-renewal and the fitness is fixed in a large fraction of runs (red, *p_a_* = *p_b_* = 0.25, *q_a_* = *q_b_* = 1/3, *a*_0_ = 1, b_0_ = 999, *r* = 1.1; blue, *p_a_* = *p_b_* = 0.15, *r* = 1.3 averaged over 1,000 runs and the percentage plotted).

A mutant SC may appear by alteration of a gene that regulates the symmetry of cellular replication. Examples of such genes include *adenomatous polyposis coli* (*APC)* [32,33] or *HUGL* [34]; both are frequently mutated in colon cancer. A mutation that changes the probability of symmetric versus asymmetric division may or may not alter the reproductive fitness of the cell. In the following, we considered these possibilities separately or in combination. We assumed that one mutant cell appears and followed the evolutionary dynamics of that mutant and its progeny; we neglected continuous production of mutants from wild-type SCs. This assumption holds if the time to fixation of such a mutant is smaller than the waiting time for production of another mutant.

The process has two absorbing states: either all SCs are normal or all SCs are mutated. We performed 1,000 simulations for each set of parameter values and plotted the fraction of simulations in which the mutant reaches fixation. A mutant without a selective advantage may take over the population by neutral drift; however, the fraction of compartments in which the mutant will dominate is small. If the mutant SC has a higher probability of self-renewal compared with the normal SC (i.e., 1 − *p_a_* − *q_a_* > 1 − *p_b_* − *q_b_*), a higher fraction of compartments is dominated by the mutant clone even in the absence of a fitness advantage (Figure 3B). As expected, mutations that increase the probability of self-renewal and improve the fitness of the SCs result in a higher frequency of fixation and in a shorter average fixation time (Figure 3C). The average time required for fixation of mutant cells decreases as the probability of self-renewal increases. For a given set of transition probabilities, the average time for fixation decreases as the population size gets smaller (Figure 4A).

**Figure 4:**
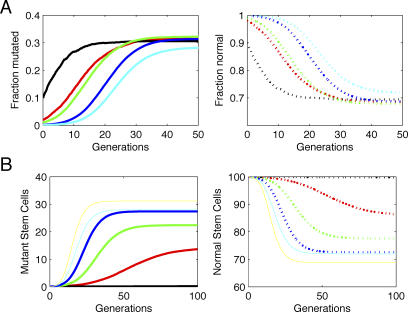
The Impact of Compartment Size *n* on the Average Time to Fixation of a Mutant Cell Based on Equation 9 (A) As the compartment size increases, the average time to fixation increases. Parameter values are *n* = 10 (black), *n* = 50 (red), *n* = 100 (green), *n* = 200 (blue), and *n* = 1,000 (cyan). For all curves *p_a_* = *q_a_* = 0.25; *p_b_* = *q_b_* = 1/3; *r* = 1.02. (B) The reproductive fitness of the mutant SCs compared with the normal SC pool plays a determining role in the evolutionary trajectory of the mutant population. A mutant with a low reproductive fitness (black) (*p_a_* = *p_b_* = 0.3; *q_a_* = *q_b_* = 1/3, *r* = 0.8, *n* = 1,000) has a low prevalence in the population, while the prevalence increases as the fitness increases (red, *r* = 1.0; green, *r* = 1.2; blue, *r* = 1.4; cyan, *r* = 1.6; yellow, *r* = 1.8). If the mutant cells have a higher fitness and increased probability for self-renewal, the probability of fixation is even higher. Each result is the summary of 1,000 simulations and the percentage plotted.

The relative fitness of the mutant SCs as compared with normal cells has a determining role in the evolutionary dynamics of the population. Mutant SCs with a reduced fitness (*r* < 1) will rarely take over the population, if the cell division properties of the mutant are the same as the wild-type SCs; as the fitness of the mutant increases, the fraction of SC compartments that will be taken over by the mutated cells increases (Figure 4B).

Let us now determine how the average fixation time depends on the probability of SC self-renewal, the size of the pool, and the fitness associated with such a mutation. A mutation that increases SC fitness requires a shorter average time for fixation in the population (Figure 5A). As the SC population size increases, the average fixation time increases nonlinearly (Figure 5B). Although mutant SCs with a reduced fitness can reach fixation in a short time, this is a highly improbable event (see Figure 4). The average fixation time for the neutral mutant is always the longest and consistent with random drift. In a similar fashion, we compute the average time to fixation, as the probability of mutant SC renewal varies from zero to two-thirds for a fixed fitness. Figure 5C shows that, as the probability of self-renewal increases (*q_a_* decreases for a fixed value of *p_a_* = 1/3), the average time for fixation of the mutant decreases. This is also true as *q_a_* increases, and therefore the probability of self-renewal decreases (Figure 5C); however, this is again an improbable event (Figure 3).

**Figure 5:**
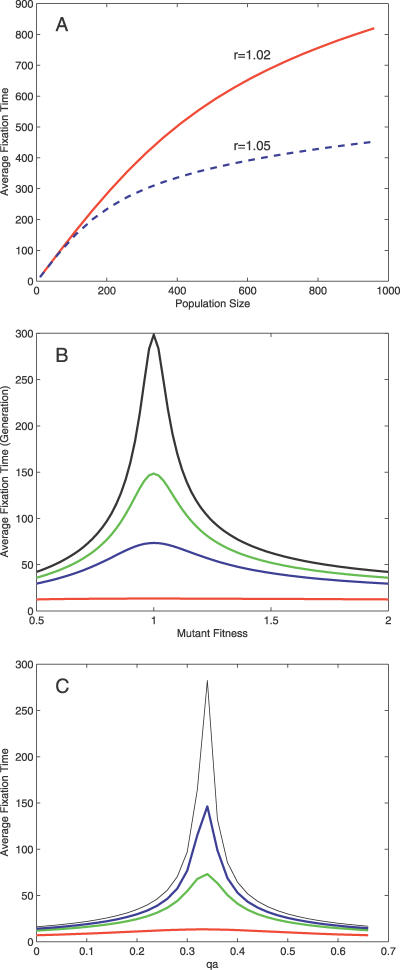
Average Fixation Time of Mutant SC The average fixation time of mutant SC depends on the relative fitness (A) (red, *r =* 1.02; blue, *r =* 1.05), population size (B), and probability of self-renewal (C) (Equation 9). The fixation time for a neutral mutant is always the longest. Although mutants with reduced fitness can reach fixation quickly, this is an improbable event. In all figures, *n* = 10 (red), *n* = 50 (green), *n* = 100 (blue), and *n* = 400 (black).

## Discussion

The field of cancer research is undergoing a transformation with the recognition that at the root of many tumors is a relatively small group of CSCs that maintains the bulk of the tumor population. The presence of these cells was initially demonstrated for hematopoietic neoplasms [35] and has been used to explain drug resistance and regrowth of the tumor when therapy is stopped [36]. However, there is increasing evidence that CSCs exist for solid tumors as well, including breast carcinoma [13], brain tumors [15], and colon carcinoma [12,16]. Studies of cells isolated from primary tumors or metastases have shown that only a fraction of the tumor population has a long-term colony forming ability or can induce tumor xenograft growth when implanted into immuno-compromised mice [12,16]. At present, it is not clear whether CSCs result from the accumulation of mutations in normal SCs or whether more committed cells acquire SC-like properties as a result of mutations. There is data to support both possibilities at least for some neoplasms, and these hypotheses are not mutually exclusive [37–39].

In this study, we explored a simple model of SC replication to illustrate the impact of changes in the probability for symmetric versus asymmetric replication on SC dynamics within a constant population. One might assume that the natural tendency of a SC is to self-renew and generate two daughter SCs. However, there is a limit on the total number of SCs in a given environment, perhaps due to a finite number of SC niches that can be occupied. Growth factor stimulation and the microenvironment within the SC niche together impose differentiation on one or both daughter cells that result from an SC division. However, the tendency of SCs to self-renew will ensure that if the SC pool is depleted (e.g., with high-dose chemotherapy), the remaining SCs can expand to occupy the available niches [40].

In evolutionary biology, reproductive fitness is defined as reproductive success within a given environment that exerts a selection pressure. In the context of cancer evolution, cells with a higher reproductive fitness either replicate faster or produce more progeny in the same time interval compared with other cells. This is often modeled as the mutant cells being selected for reproduction with a higher probability compared with the normal cells. Our model shows that mutations that increase the probability of SC self-renewal (larger 1 − *p_a_* − *q_a_*) give the mutant cell an advantage even though the mutant may not be selected for reproduction more often than a normal SC. This can be deduced from Equations 1 and 2 since whenever 1 − *p_a_* − *q_a_* > 1 − *p_b_* − *q_b_,* the probability of fixation of the mutant increases. If we consider a mutation that does not confer a selective advantage to the cell (*r* = 1), the ratio of the transition probabilities reduces to:

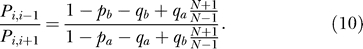



Thus, as the probability to generate two daughter cells, *q_a_,* decreases, while all the other parameters remain fixed, the ratio of the transition probabilities decreases and makes fixation of the mutant more likely. Moreover, mutations such as those in *APC* that increase both the probability of self-renewal and the probability that the mutant is selected for reproduction (i.e., increase *r*) give an even higher advantage to the cell, and the fixation time decreases while the probability of fixation increases.

The risk of cellular transformation is in part dependent on the population of cells at risk as well as on the mutation rate. While the number of normal SCs appears to be tightly regulated, mutations in critical genes may reduce the responsiveness of SCs to environmental controls, and their population can expand. Alternatively, mutations in genes such as *Partner of Inscuteable (PINS)* [41], *lethal giant larvae (LGL)* [42], and *HUGL-1* that regulate the symmetry of SC replication may lead to expansion of this cell pool. Mutations in these genes are associated with the development of tumor-like tissue in model organisms [42,43]. In colonic crypts, *APC* normally controls SC reproduction by inducing the degradation of *β-catenin* [44]. Therefore, mutations in *APC* might lead to an expansion of the SC pool at the base of the colonic crypt. In addition to inhibiting *β-catenin, APC* also regulates the (a)symmetry of stem cell division in *Drosophila* [45]. It is not known whether *APC* mutations within colonic crypt SCs increase the probability of symmetric division with SC expansion. However, patients with familial adenomatous polyposis develop aberrant crypt foci that are associated with an increased risk of colonic adenomas as well as colon cancer [46]. With the identification of CD133 as a marker for colon CSCs [12,16], it would be interesting to determine whether aberrant crypt foci are enriched for crypt SCs and what additional mutations are present in these cells apart from *APC*. Further, there is evidence that prostaglandins may be necessary for SC growth and asymmetric replication [47]. Perhaps this may in part explain the reduced risk of colon cancer associated with the use of nonsteroidal anti-inflammatory drugs [48] and other COX-2 inhibitors.

In summary, SCs may in principle replicate via three pathways to expand their own population or produce differentiated daughter cells. Mutations in genes that increase the probability of self-renewal provide an additional facet for the selective advantage of tumor SCs. The identification of additional genes that regulate the (a)symmetry of SC replication will expand our understanding of the growth of the tumor SC clone and possibly identify novel targets for cancer therapy.

## Abbreviations

*APC*: *adenomatous polyposis coli*
CSC: cancer stem cell
SC: stem cell
