# Correction: (A)Symmetric Stem Cell Replication and Cancer

**DOI:** 10.1371/journal.pcbi.0030083

**Published:** 2007-04-27

**Authors:** David Dingli, Arne Traulsen, Franziska Michor

In *PLoS Computational Biology,* volume 3, issue 3: doi: 10.1371/journal.pcbi.0030053

An author's name was misspelled as Fransizka Michor and should read:

**Franziska Michor**

